# “The Laryngectomee Guide” is available now in Portuguese^☆^

**DOI:** 10.1016/j.bjorl.2019.06.007

**Published:** 2019-07-24

**Authors:** Itzhak Brook

**Affiliations:** Georgetown University Medical Center, Washington, USA

Dear Editor,

I am happy to announce that “The Laryngectomee Guide” is available now in Portuguese. The translation was supervised by Drs. Aline L. Freitas Chaves, José Guilherme Vartanian, Rogério A. Dedivitis, and Luiz P. Kowalski and is available (FREE) at: https://goo.gl/aBXGD8.

The paperback edition can be obtained at https://amzn.to/2KZbfbq.

The American Academy of Otolaryngology Head and Neck Surgery has made the English edition available for free download on their website: http://www.entnet.org/content/laryngectomee-guide.

The Guide provides practical information that can assist laryngectomees and their caregivers with medical, dental and psychological issues. It contains information about side effects of radiation and chemotherapy, methods of speaking, airway, stoma, and voice prosthesis care, as well as eating and swallowing, medical, dental and psychological concerns, respiration, anesthesia and travel guidelines.^1^

I hope that you will find the Guide helpful for you and your colleagues as well as your patients.

Thanks.

## Conflicts of interest

The author declares no conflicts of interest.
